# Multimodal Bio-Inspired Tactile Sensing Module for Surface Characterization †

**DOI:** 10.3390/s17061187

**Published:** 2017-05-23

**Authors:** Thiago Eustaquio Alves de Oliveira, Ana-Maria Cretu, Emil M. Petriu

**Affiliations:** 1 School of Electrical Engineering and Computer Science, University of Ottawa, Ottawa, ON K1N 6N5, Canada; petriu@uottawa.ca; 2 Department of Computer Science and Engineering, Université du Quebec en Outaouais, Gatineau, QC J8X 3X7, Canada; ana-maria.cretu@uqo.ca

**Keywords:** tactile sensing, MARG system, MEMS, robotic probe, surface profile classification

## Abstract

Robots are expected to recognize the properties of objects in order to handle them safely and efficiently in a variety of applications, such as health and elder care, manufacturing, or high-risk environments. This paper explores the issue of surface characterization by monitoring the signals acquired by a novel bio-inspired tactile probe in contact with ridged surfaces. The tactile module comprises a nine Degree of Freedom Microelectromechanical Magnetic, Angular Rate, and Gravity system (9-DOF MEMS MARG) and a deep MEMS pressure sensor embedded in a compliant structure that mimics the function and the organization of mechanoreceptors in human skin as well as the hardness of the human skin. When the modules tip slides over a surface, the MARG unit vibrates and the deep pressure sensor captures the overall normal force exerted. The module is evaluated in two experiments. The first experiment compares the frequency content of the data collected in two setups: one when the module is mounted over a linear motion carriage that slides four grating patterns at constant velocities; the second when the module is carried by a robotic finger in contact with the same grating patterns while performing a sliding motion, similar to the exploratory motion employed by humans to detect object roughness. As expected, in the linear setup, the magnitude spectrum of the sensors’ output shows that the module can detect the applied stimuli with frequencies ranging from 3.66 Hz to 11.54 Hz with an overall maximum error of ±0.1 Hz. The second experiment shows how localized features extracted from the data collected by the robotic finger setup over seven synthetic shapes can be used to classify them. The classification method consists on applying multiscale principal components analysis prior to the classification with a multilayer neural network. Achieved accuracies from 85.1% to 98.9% for the various sensor types demonstrate the usefulness of traditional MEMS as tactile sensors embedded into flexible substrates.

## 1. Introduction

Recognition of objects by touch is one of the first steps to enable robots to help humans in their everyday activities. Many applications such as health and elder care, manufacturing, and high-risk environments involve tasks in unstructured environments that require robots to handle various types of objects, made of different materials, that can be situated out of the field of view of the robot or partially obstructed. In order to cope with these situations, improvements must be made both in sensing technologies enabling the robot touch and in the capability of robots to make sense fast and efficiently of perceived data. 

Similar to humans that recognize the properties of an object by touching it, in order to manipulate objects efficiently, robots need to first be able to identify them based on their properties. Object identification by touch can be divided into the identification through static or dynamic touch. In static touch, the tactile sensing apparatus establishes contact with an object and collects data while the object is in touch with the probe; the apparatus and explored object are not moving during the sensing process. This approach can, for instance, gather data on temperature or information on the local geometry of the object. In dynamic touch, the tactile apparatus gathers data while the sensors slide over the object’s surface, similar to human exploration of surfaces by lateral motion. Klatzky [1] observed that people use a lateral motion with one or more fingers, that may move more quickly or more slowly, using either a rubbing in circle or a short sweep in order to detect an object’s roughness. The invariant aspect of this exploratory movement is the fact that the skin moves tangentially across the local of the surface. Similarly, a sliding movement over an object executed by a robot finger can enable robots equipped with appropriate sensors to collect information about the roughness of its surface and help identify its properties.

Although several efforts have been made on the development of robotic touch and related sensors that can detect multiple types of stimuli, the current generation of MEMS sensors have not yet been extensively explored while embedded in flexible materials, and in the context of multimodal sensing. Very few solutions in the literature use more than one type of sensor to collect tactile information. Moreover, the existing solutions fail to provide an integrated approach for multimodal tactile module design, in particular for the placement of sensors within compliant structures. These are some of the issues that the current work attempts to tackle.

This paper focuses on the issue of surface characterization through sliding motions, performed by a linear motion carriage and by a robot finger composed of three motors, both equipped with a bio-inspired multimodal tactile module. Taking inspiration from the function and the organization of mechanoreceptors in the human skin on one side and from the hardness of human skin, on the other side a novel tactile module is built, comprising a 9-DOF MEMS MARG (Magnetic, Angular Rate, and Gravity) system and a deep MEMS pressure (barometer) sensor, both embedded in a compliant structure that mimics the hardness of human skin. When the module’s tip slides over a surface, the MARG unit vibrates and the deep pressure sensor captures the overall normal force exerted.

The module is evaluated in two experiments. The first experiment compares the frequency content of the data collected in two setups: in the first setup, the module is mounted over a linear motion carriage that slides four grating patterns at constant sliding velocities. In this setup, the orientation of the module with respect to the grating patterns is also kept constant. This is an ideal setup that evaluates the basic capabilities of the tactile module and that is similar to the testing conditions in most of the current work in the literature. In the second setup, the module is carried by a robotic finger that keeps contact with the same grating patterns while performing a sliding motion similar to the exploratory motion employed by humans to detect object roughness. In this setup, the orientation, velocity and pressure exerted by the module are not kept constant. This sort of setup that characterizes real-world applications, in which the robot needs to deal with unknown objects and in which a constant orientation, velocity and pressure are almost impossible to achieve, allows evaluating the capabilities of the tactile module in realistic conditions. The second experiment shows how localized features extracted from the data collected by the robotic finger over seven synthetic shapes can be used to classify them and therefore enabling the robot to characterize them. The method employed to distinguish them consists on applying multiscale principal components analysis prior to the classification with a multilayer neural network.

This paper builds on our previous work in [2]. The paper now includes a detailed description of a novel bio-inspired tactile module, accompanied by a thorough evaluation on tasks related to dynamic surface characterization. The additional evaluation aims at analyzing the module’s response on gratings exploration tasks both in ideal conditions (constant orientation as encountered in most of the work in the literature) and in real conditions, where the sensor is attached to a robotic finger and no constraints are imposed. The contributions of this paper are: (1) the design of a tactile module inspired from biology, mimicking the mechanoreceptors’ placement, function and the hardness of skin within the compliant structure that embeds the sensors; (2) the comparison of data collected in frequency and time domains on an orientation-constrained linear setup to the one sensed while performing the exploration by a 3-DOF robotic finger; and (3) the classification of data collected by the robotic finger over synthetic shapes with localized features. 

The paper is organized as follows: The next section discusses relevant work in the literature. The material and methods employed in the experimental setup are detailed in Section 3. Section 3.1 presents the bio-inspired tactile module. The response of the sensors embedded in the module to ridged surfaces (grating patterns) is presented in Section 3.2.1. Section 3.2.2 presents our approach to the problem of shape discrimination from multi-sensory data collected during a sliding motion of a robot finger. Finally, insights on future work and final considerations are described in Section 4.

## 2. Literature Review

The design of tactile sensors and the interpretation of the data gathered by such sensors is the subject of vast literature. Most of the solutions in the literature propose a single sensing technology, such as pressure/normal forces sensors, accelerometers, fiber optic and other optical sensors, and microphones to dynamically acquire tactile data for texture identification [3].

The most explored sensing modality in tactile sensing is pressure or force sensing. In [4,5], a tactile sensor array based on MEMS sensors embedded in a polymeric packaging and inspired from the SA1 innervation density in humans, is investigated for roughness encoding. A time-frequency analysis on pairs of tactile array outputs yields 97.6% discrimination accuracy with a k-nearest-neighbor (k-NN) classifier. The authors of [6] perform tactile texture recognition with a 3-axial force MEMS integrated in artificial finger that slides on a surface. Supervised classification methods are used to discriminate fine textures over 10 kinds of paper.

In [7], Jammali and Sammut present a system where textures are distinguished by the presence of different frequencies in a signal collected from a fingertip equipped with randomly distributed strain gauges and polyvinylidene fluoride (PVDF) films embedded in silicone. The Fourier coefficients of the signal are used to train a series of classifiers (i.e., naive Bayes, decision trees, and naive Bayes trees). The classifiers achieved an accuracy 95 ± 4% on materials such as carpet, flooring vinyl, tiles, sponge, wood, and woven mesh.

Another category of solutions to collect tactile information for surface identification is based on the use of accelerometers. Dynamic exploration tasks such as the identification of shapes and textures benefit from the data collected by such sensors, both in the cases where the sensor is static and the object is moving [8] and in the scenarios where the sensor is moving and the object is static [9]. A tactile probe in form of a small metallic rod with a single-axis accelerometer attached to its tip is employed to classify surfaces based on their mean, variance, and higher order moments using a support vector machine in [10]. A training data set is collected over 28 different indoor and outdoor surfaces while the test surface is rotated by a turntable, and a neural network achieves a surface recognition rate of 96.7% based on 1 s of data. The authors demonstrate as well that similar results can be achieved without the need for ground truth or the actual number of surfaces using Dirichlet process mixture models, a Bayesian nonparametric approach. Due to the use of a turntable, such a solution cannot be readily applied in interactions between a robot and an object. Considerations about the changes in orientation, position, and velocity of the sensor relative to the object are obstacles to the direct application of such a setup in active sensing applications. The same idea of a metallic rod dragged along a surface to be identified is also explored in [11]. In [11], eight features in time and frequency domains are used for classification using a neural network and a recognition rate of 90.0–94.6% for a 1- and 4-s time-window is achieved for ten types of indoor and outdoor surfaces. An unsupervised classification recognition rate of 74.1% for 1-s time windows of terrain data is achieved in [12]. A three-axis accelerometer captures vibro-tactile feedback as a robot is performing a scratching motion in [13] and the collected data over 20 surfaces is used to recognize the surface material using machine learning approaches.

Another category of tactile sensing solutions uses optical and fiber optical sensors. The tactile sensor of De Maria et al. [14] is based on a matrix of LED-phototransistor couples embedded in a deformable elastic layer in the form of a hemisphere. The deformable layer transduces external forces and torques into local deformations which produce a variation of reflected light intensity and of the photo-current flowing in the photodetectors. A model of the same sensor based on extended Kalman filter allows to reconstruct the position and orientation of the surface in contact with a rigid object [15]. The friction coefficient is then estimated based on the contact plane position and orientation information together with the contact force vector measured by the sensor. A slippage control algorithm for the same sensor is proposed for manipulation tasks in [16]. Chorley and coworkers’ sensor [17], based on the morphology of the fingertip skin in encoding tactile edge information, comprises a thin flexible rubber skin with structural details and encases a clear, compliant polymer melt blend. Markers on the internal structural details of the rubber skin are tracked by an embedded CCD, illuminated by four infrared LEDs and enable detection of surface deflections. The same sensor is used to detect object edges in the context of a robotic contour following task in [18]. The authors of [19] detect contact and control forces during fine manipulation with the help of four fiber optical sensors embedded in an exoskeletal robot finger. Fiber Bragg gratings ensure a high strain sensitivity and immunity to electromagnetic interference. 

In [20], an artificial finger equipped with a microphone to detect frictional sound is employed to quantify texture. Recordings of a series of artificial surfaces were made to create a library of frictional sounds for data analysis and were mapped to the frequency domain using fast Fourier transforms. Features such as modal frequency and average value were calculated to analyze the data and compared with attributes generated from principal component analysis. Classification of the recordings using k-nearest neighbors obtained a high accuracy for PCA data. The idea of using a microphone is also exploited in [21,22]. The authors developed a microphone based texture sensor and a hardness sensor that measures the compression of the material at a constant pressure. Raw-sensor output is conveyed to a self-organizing map that represents after training the textural properties of the explored materials.

All standalone sensing technologies are associated with specific drawbacks. Magnetic sensors can only be used within environments with low magnetic field variations. Fiber optic sensors are expensive and difficult to integrate in tactile modules due to the bending losses in the fiber routing [14]. CCD camera solutions involve generally a high cost and weight, while the loss of light due to microbending or chirping causes distortion is the measured data [23]. To alleviate the drawbacks associated to any one single technology, a possible solution is to capitalize on the use of multiple sensor technologies for recuperating tactile information [8,24,25,26]. A biomimetic fingertip containing three accelerometers and seven force sensors in two layers of polyurethane [27], is employed to discriminate six fabrics and an aluminum plate by comparing the differences in their surface texture in [28]. An input signal is computed by calculating the covariance of two adjacent accelerometers and a neural-network classifier using as features the variance and power of the accelerometer signal is used to discriminate the seven textures. The same fingertip distinguishes seven wood samples in [24], by training a neural-network based on three features calculated from the covariance signal of two adjacent accelerometers in the fingertip, namely the mean and variance of the approximate signal, and the energies of the detailed signal. The covariance signal is transformed using Discrete Wavelet Transform and a 65% success rate is reported for classifying the wood samples. The authors of [29] use multimodal tactile sensing (force, vibration and temperature) and program a robot hand to make exploratory movements similar to those humans make when identifying objects by their compliance, texture, and thermal properties. The proposed Bayesian exploration algorithm is augmented with reinforcement learning to enable the evolution of internal representations of objects according to cumulative experience. The robot is able to correctly identify 10 different objects on 99 out of 100 presentations. A data-driven analysis to the problem of sensor selection in the contour following for shape discrimination task is presented in [26]. Data collected from the motors, an inertial measurement unit, and a magnetometer attached to a 4-DOF robotic finger, during the exploration of seven synthetic shapes are analyzed using principal component analysis and a multilayer perceptron neural network is trained to classify the shapes. Kroemer and coworkers’ [25] tactile sensor consists of a compliant pin that makes contact with a surface and a capacitor microphone that detects the vibrations of the pin. A multisensory data fusion algorithm combines readings from the pin with vision data collected with a camera to classify rich-textured surfaces.

This paper tackles the dynamic tactile surface characterization problem using a novel bio-inspired tactile module that uses traditional MEMS sensors embedded in a compliant substrate mimicking the function and placement of mechanoreceptors as well as the hardness of the human skin. In particular, a 9-DOF MEMs MARG (Magnetic, Angular Rate, and Gravity) module and a MEMs pressure (barometer) sensor are positioned so that when the tip of the module is rubbed over a surface, the MARG unit vibrates and the deep pressure sensor captures the overall normal force exerted. The experiments show that the traditional MEMS sensor can detect the stimuli generated by grating patterns used in other sensors’ validation [4], even when the sensors are embedded in a truly compliant structure. This paper also shows the performance of the module in realistic interaction scenarios between a robotic finger and an unknown surface in which the orientation, velocities and forces are not kept constant. As it is demonstrated in the experimental part of this paper, the signals measured in such conditions can be used to classify surface profiles.

## 3. Bio-Inspired Tactile Sensor Module and Surface Characterization Experiments

### 3.1. Bio-Inspired MEMS Based Tactile Module

**Figure 1:**
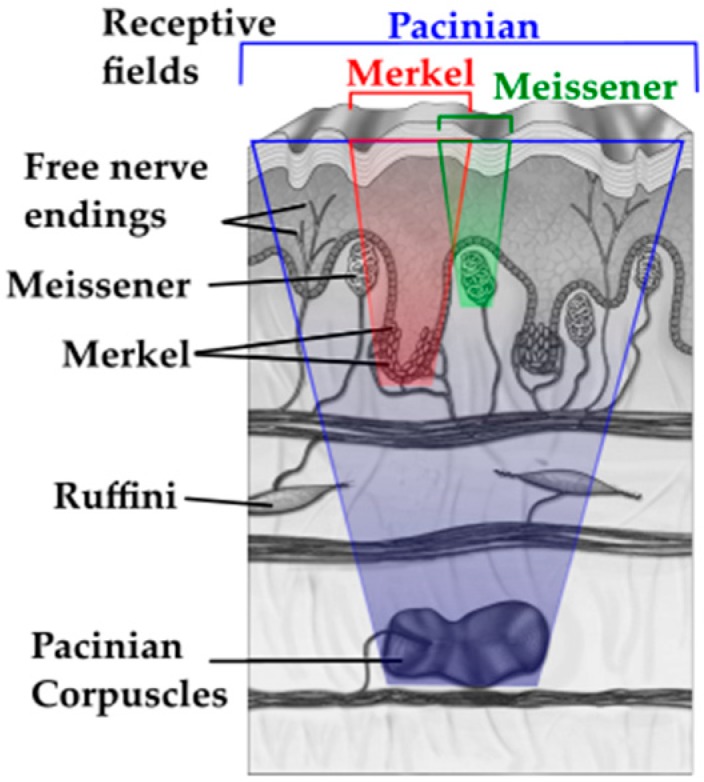
Human skin mechanoreceptors, adapted from [32]; Pacinian corpuscle receptive field in blue; Merkel receptive field in red; and the Meissner receptive field in green.

The tactile module presented in this work draws inspiration from the human tactile system. According to Loomis and Lederman [30], tactile perception refers to sensing capabilities mediated by cutaneous stimulation, involving a physical contact between an object and the surface of the skin that encases the mechanoreceptors [23]. The cutaneous stimuli sensed by tactile receptors embedded in the skin are interpreted into information about topology, texture, contact force, elasticity of the touched object’s surface, and other features. The skin on the human hands contains four types of cutaneous mechanoreceptors [31,32], Meissner’s corpuscles, Merkel disks, Pacinian and Ruffini corpuscles, as illustrated in Figure 1.

Merkel disks and Ruffini corpuscles are slow adapting, i.e., detect constant stimuli (e.g., constant pressure or skin stretch). Merkel disks can also detect initial contact. Meissner and Pacinian are fast adapting receptors able to detect short pulses, such as the initial contact. Meissner and Merkel are located in shallow levels, close to the skin surface, while Pacinian and Ruffini are present in deeper levels of the human skin. While the exact role, function and properties of each of these receptors are not yet completely understood, Table 1 summarizes aspects accepted in the current literature from neuroscience and experimental psychology [23,32,33].

Mechanoreceptors in the human skin work in synergy to enable the correct perception of different stimuli that emerge from manipulation of objects or from haptic exploration. For example, in the context of edge detection, Meissner’s corpuscles sense small sharp borders in dynamic touch tasks, while Merkel disks sense the same information during static touch. Larger surface features are sensed by Pacinian and Ruffini corpuscles due to their large receptive fields. Mechanoreceptors are sensitive to mechanical stimuli around the skin’s surfaces on areas ranging from 9 to 60 mm^2^. The receptive fields of such cells are combined and superimposed to achieve two-point discrimination of as low as 2 mm on human hands [23]. Some mechanoreceptors are more specialized in static exploration, while others in dynamic scenarios. Dynamic tactile data are gathered when skin ridges on the surface slide in contact with explored objects. Properties such as stickiness, smoothness, friction, roughness, slippery can be hardly estimated by static probing [34].

**Figure 2:**
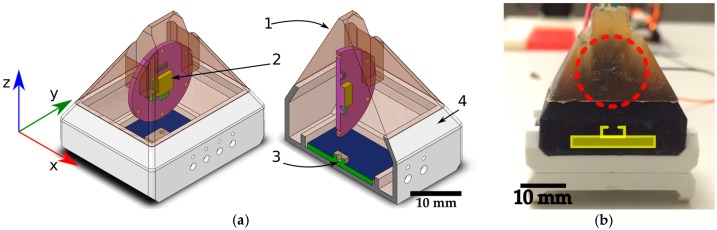
(**a**) Tactile module: 1, pyramidal compliant structure; 2, MARG system on printed circuit board (PCB), the MARG system land grid array package is highlighted in yellow; 3, deep pressure sensor (barometer ventilation window); and 4, supporting collar. (**b**) Front view of the tactile probe: the MARG system is embedded under the red circle; the pressure sensor is under the yellow overlay in the black 3D printed collar. A side view of the module can be seen in Figure 3a.

The focus of the bio-inspired tactile module developed in this article is to gather dynamic tactile data. It integrates and organizes conventional MEMS sensors embedded in a compliant structure in a manner similar to the mechanoreceptors in the human skin, while focusing on the study of dynamic tasks. It takes inspiration from the type of mechanoreceptors present in glabrous skin, from their two-layered organization (Figure 1) and from their functional relationships. Therefore, it consists of a shallow 9-DOF MARG (Magnetic, Angular Rate, and Gravity) sensor, a flexible compliant structure and a deep pressure sensor, as shown in Figure 2. It implements the deep pressure sensing functionality by using a MEMS barometer embedded in bottom of the pyramidal compliant structure. This component of the tactile module reproduces the functions of Pacinian corpuscles in sensing deep pressure and pressure changes (Table 1). The compliant pyramidal structure conducts forces applied to the shallow sensors in the tip of the structure to the barometer ventilation window (Figure 2a(3)). The shallow sensors implemented by a MARG system measure vibrations (accelerations, angular velocities and changes in the magnetic field surrounding the module) (Figure 2a(2)) on the higher levels of the module and emulate the functionalities of Merkel cells and Meissner’s corpuscles.

The module in this article does not focus on the tactile imaging that is usually performed by pressure sensitive tactile arrays in static tactile exploration, but some of the sensors embedded in the module could play the role of mechanoreceptors specialized in static tactile functions, for example the gravity and magnetic field sensors.

The compliant structure connects the shallow and deep sensors (Figure 2a(1)). This structure performs the role of the receptive field, refining the spatial resolution of the shallow sensors and conducting the forces applied on the tip of the pyramidal structure to the deep pressure sensor. The pyramidal structure tries to mimic the area corresponding to the intersection between the receptive fields of one Pacinian (deep pressure sensor), one Merkel cell, one Meissner's corpuscle and one Ruffini nerve ending. The PCB on which the MARG system is mounted helps guiding pressures in the fingertip to the deep pressure sensor. The compliant structure deformation makes the shallow sensors vibrate when the module’s tip slides on external surfaces. The compliant structure in the prototype has shore 20 A hardness, similar to the human skin in the index fingertip pad [35]. Structures with hardness lower than shore 20 A tend to degrade fast and would need to be repaired often, harder structures (e.g., hardness shore 70 A) would limit the deformability of the module vibration of the MARG system. The flexible structure also adds compliance to the module. The authors of [36] state that compliance makes sensing more efficient by reducing the control precision required to maintain gentle contact with an object, avoiding sharp forces during exploration, and increasing the robustness of the sensors.

The proposed bio-inspired tactile module capitalizes therefore on a combination of various sensors: The *accelerometer* provides information on the orientation of the shallow sensors relative to a gravity frame. It is also useful to detect light touch and deformation on the tip of the compliant structure. Dynamic exploration tasks such as the identification of shapes and textures benefit from the data collected by accelerometers. This component of the tactile module emulates a behavior resembling the one of Merkel cells within human skin. The 3-DOF *angular rate sensor (gyroscope)* embedded in the same integrated circuit (MARG unit) of the accelerometer collects data about the rate of vibrations onto the shallow sensors (angular velocities). The rate of deformation that occurs on the tip of the module is related to the angular rate of the shallow sensors. The measured data acquired by this sensor simulate the one recuperated by Meissner’s corpuscles within the glabrous skin. The 3-DOF *magnetometer* measures the orientation of the shallow sensors, thus the deformation of the module’s tip, relative to a reference magnetic field that can be either the earth’s magnetic field or a local magnetic field induced by permanent magnets. Permanent magnets can be employed as local reference frames while measuring stretch or deformation of flexible substrates. However, relying solely on magnetic measurements is not an ideal scenario for tactile sensing due to the magnetic interference when touching ferrous objects or when working in ferrous environments. Machine learning systems and other algorithms applied only on magnetic measurements could be misled by the presence of the unexpected ferrous object. This is one of the reasons why it is desirable to use complementary sensors, for example accelerometer, gyroscope and magnetometer in the same integrated circuit, as in the current work. The capability of this component of our tactile module to provide form and stretch information mimics the functionality of Ruffini endings. Finally, the role of the Pacinian corpuscles is performed by the deep pressure sensor realized by a MEMS barometer encased in polyurethane on the bottom of the compliant structure. The height of the compliant structure from the tip to the ventilation window Figure 2a(3) of the barometer is 28 mm. This sensor collects data on the pressure of un-localized touch around the tip area of the module and high frequency changes in pressure.

The 9-DOF MARG system used for the experimentation is the STMicroelectronics© LSM9DS0 composed of a triple-axis accelerometer, a triple-axis gyroscope, and a triple-axis magnetometer. The pressure sensor (i.e., MPL115A2 from Freescale Semiconductor©,) sits on the bottom of the black 3D printed collar that holds the compliant structure. The flexible rubber from the compliant structure is made of VytaFlex© Shore 20 A hardness. The microcontroller serving as an interface between the computer that collects and analyzes the data from the sensors is a Freescale Semiconductor© MK20DX256VLH7 Cortex-M4 at 72M Hz. It is connected to the sensors through a two-wire interface (TWI) at 400 K Hz. The microcontroller connects to the computer through a USB Serial interface. The data acquisition software is developed using the Robotic Operating System (ROS). The computer running ROS accesses the data from the sensors through the interface microcontroller at 440 Hz. Table 2 shows the details (measurements range, frequency range, and sensors resolution) for the MARG system and the deep pressure sensor used in this work.

### 3.2. Experimental Setup

Two experiments were performed with the tactile module introduced above, the first one exploring the capability of the sensor to sense gratings on surfaces in a linear motion setup and when it is attached to the tip of a robotic finger. The grating patterns are inspired by the experiments in [39] and employ the same dimensions as in [4], namely the same ridge width and height and groove width (i.e., distance between ridges). The second experiment focuses on the application of the module to the exploration and identification of coarser tactile profiles. 

#### 3.2.1. Experiment 1: Sensors Response to Ridged Surfaces (Grating Patterns)

During this experiment, the tactile module is tested in two setups to evaluate its capability to detecting ridges on surfaces and their spatial distribution. 

**Figure 3:**
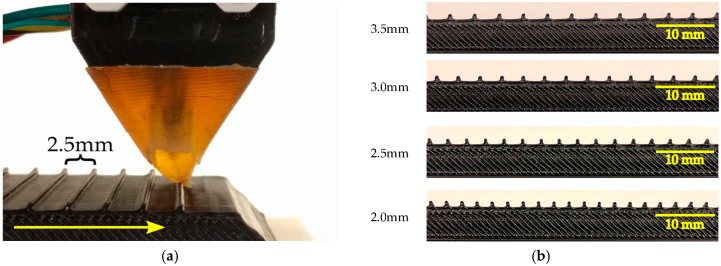
Sliding apparatus and gratings patterns: (**a**) side view of the tactile module in the beginning of the stimulation; and (**b**) grating patterns with a groove width varying from 2.0 mm to 3.5 mm; the depth of the grooves is 1 mm and the thickness of the ridges is 0.6 mm.

In the first setup, the module is fixed over a linear motion setup. The purpose of this setup is to evaluate the sensors and compliant structure’s response to regularly spaced ridges on a surface while keeping the module in a steady orientation relative to the grating pattern surface. The tactile module is mounted above a linear motion actuator carriage where gratings with different patterns are fixed to stimulate the tactile module deforming its fingertip while sliding under it. Figure 3a shows a close-up view of the tactile module and the 2.5 mm groove width grating pattern in the beginning of the stimulation. The sliding motion occurs along the sensor’s *x*-axis. The fingertip is slightly deformed due to the contact with the first ridge. Figure 3b shows the grating patterns with a groove width varying from 3.5 mm to 2.0 mm. The depth of the grooves is 1 mm and the thickness of the ridges is 0.6 mm. The grating pattern with 3.5 mm groove width has 20 ridges; the ones with 3.0 mm, 2.5 mm, and 2.0 mm groove have 23, 26 and 31 ridges, respectively. The length of the 3D printed grating patterns is 100 mm. The height of the layers in the 3D printed patterns is 0.1 mm. To minimize the impact on measurements, the layers were deposited such that the module did not cross layers during the exploratory movement.

During experimentation, the patterns were approximately aligned to the *x*-*y* plane of the sensor. This was done on purpose, since keeping a steady pressure should not be an obstacle to identifying the frequencies induced by the patterns. For instance, monkeys do not keep a steady pressure while performing surfaces exploration [39].

The experiment and data acquisition started with the module unloaded, i.e., not in contact with the grating pattern, and then the linear platform carrying the grating pattern brought it to contact the tip of the module. The contact of the pattern and tip generates a small load that is detected by the sensors embedded in the compliant structure. When the sensing module encounters the ridges of the grating pattern, larger loads are driven into the structure of the module and exerted on the sensors.

The sliding stimulus occurred with two translational velocities (15 mm/s and 30 mm/s, respectively), and were chosen in accordance to the ones used in [4]. The velocities, directions and starting position of the gratings related to the module were the same for all gratings. The travel distance for the linear carriage was 130 mm, ensuring that the entire length of the grating pattern passed under the modules tip. The dynamic stimulations from the first to last ridge lasted from 5.6 s to 2.8 s depending on the velocity employed. By the end of the sliding motion the module was unloaded since the entire grating pattern slid under the tip and the contact was interrupted.

The authors of [40] suggest that human beings perceive roughness sensation as the change of frequency detected by Meissner’s corpuscles in relationship with their hand movements and the physical properties of roughness of materials. According to the same authors, given velocity v, the frequency of stimuli f, which are generated in a point of finger, can be expressed by f=v/Δp, where f is the frequency in Hz, v is velocity measured in mm/s and Δp is the groove width plus the ridge width. Assuming the velocities of 15 mm/s and 30 mm/s, the expected frequencies generated in a point of the tip of the tactile module for the different grating patterns are defined in Table 3. The information in this table will be used later to validate the response of our tactile module.

**Figure 4:**
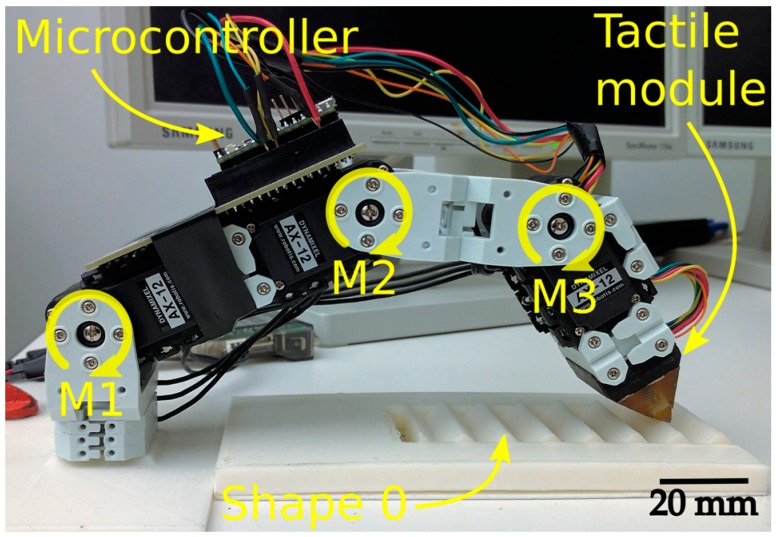
Robot finger composed of three motors: M1 is the “bottom” motor; M2 is the “middle” motor; and M3 is the “top” motor. The microcontroller is attached on top of Motors M1 and M2.

In the second setup, the module was mounted on the tip of a 3-DOF robotic finger as an end effector. The finger prototype used to perform the sliding motions can be seen in Figure 4. It contains three Robotis Dynamixel AX-12A robot actuators labeled M1 (bottom), M2 (middle) and M3 (top). The motors are controlled by a ROS node joint position controller. Each joint adjusts its position to minimize the difference between its current position and the one in the prerecorded movement. The prerecorded movement started with the finger extended and the tactile module touching the ramp on the beginning of a shape without gratings (a blank pattern). The finger carried the module through the extension of the grating patterns keeping contact between module and pattern with unconstrained fingertip orientation and pressure. The average velocity along grating pattern was 33 mm/s.

**Figure 5:**
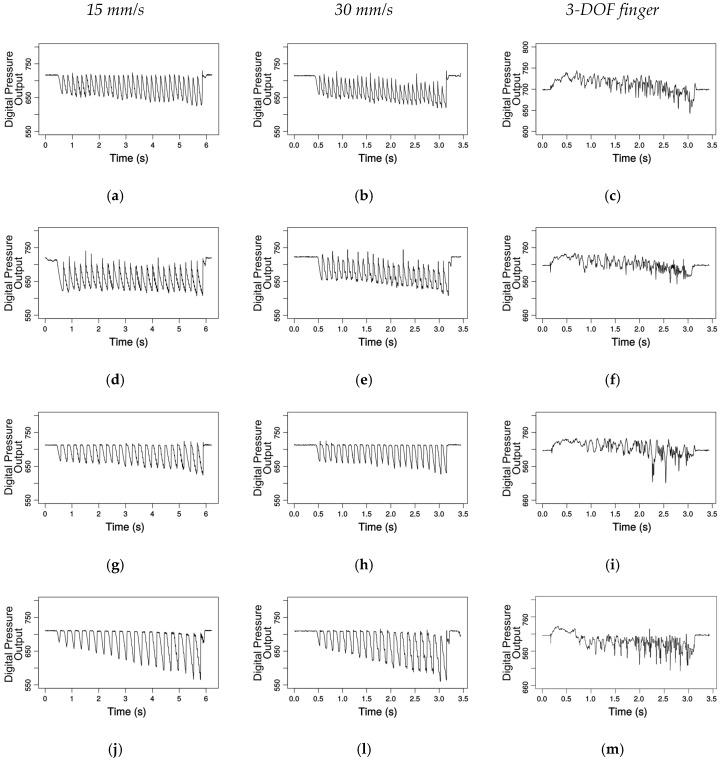
Pressure response for various groove widths of the gratings pattern: (**a**–**c**) 2 mm (31 ridges); (**d**–**f**) 2.5 mm (26 ridges); (**g**–**i**) 3.0 mm (23 ridges); and (**j**–**m**) 3.5 mm (20 ridges).

Figure 5 shows the pressure response captured by the module for the grating patterns explored by the linear motion setup for various speeds and by the robotic finger. The first two columns show the data gathered under a velocity of 15 and 30 mm/s, respectively. The last column shows the pressure response for the exploration performed by the robotic finger using an average velocity of 33 mm/s. The rows of graphs show the data collected for the gratings from the 2 mm groove width in the first row to the 3.5 mm in the bottom row. The graphs from the first two columns show clear valleys in the raw output of the pressure sensor corresponding to the ridges in the grating pattern; as the pressure inside the collar increases, the digital output of the pressure sensor decreases. The deformation of the fingertip increases the pressure inside the collar sensed by the deep pressure sensor. The deformation and the rate at which the deformation occur are sensed by the gravity and angular velocity MARG system. These measurements are independent of pressure. The patterns were slightly inclined relative to the module, i.e., the *z*-axis from the module was not perfectly normal to the grating pattern plane; this aspect is more noticeable on the 3.5 mm plots. These small misalignments are not an issue when detecting the number of ridges in the patterns and should not affect the frequency of the stimuli detected by the various sensors.

The first two columns show the data gathered under a velocity of 15 and 30 mm/s, respectively. The last column shows the pressure response for the exploration performed by the robotic finger using an average velocity of 33 mm/s. The rows of graphs show the data collected for the gratings from the 2 mm groove width in the first row to the 3.5 mm in the bottom row. The graphs from the first two columns show clear valleys in the raw output of the pressure sensor corresponding to the ridges in the grating pattern; as the pressure inside the collar increases, the digital output of the pressure sensor decreases. The deformation of the fingertip increases the pressure inside the collar sensed by the deep pressure sensor. The deformation and the rate at which the deformation occur are sensed by the gravity and angular velocity MARG system. These measurements are independent of pressure. The patterns were slightly inclined relative to the module (within ±2 degrees), i.e., the *z*-axis from the module was not perfectly normal to the grating pattern plane; this aspect is more noticeable on the 3.5 mm plots. This small misalignment is not an issue when detecting the number of ridges in the patterns and should not affect the frequency of the stimuli detected by the various sensors.

Well-structured setups as the linear motion presented here and in [4,39] are useful to validate and analyze the feasibility of application for tactile sensors, but the assumptions of constant velocity, of constant pressure (or regular increments in pressure) as well as of constant orientation of the sensing apparatus limit the applicability of such sensors in more elaborated robotic applications. The third column of Figure 5 illustrates a scenario closer to real-world applications. The last link of the 3-DOF robotic finger was not constrained to have constant orientation, velocity or pressure. This is the case of almost all real situations involving the exploration of unknown surfaces by autonomous robots. The only constrain in the prerecorded trajectory was the uninterrupted contact between fingertip and grating pattern. As one can notice in the last column of Figure 5, different from the linear motion setup, the data collected by the 3-DOF finger did not present noticeable valleys in pressure. This is due to the changes in orientation and velocity of the fingertip as well as to the variations in force exerted by the fingertip along the movement. Localized features in the collected signals can still be used as discriminative inputs for classification systems.

In order to analyze the frequency content of the signals collected by the sensors in each setup, the raw discrete signals z were subject to a non-normalized discrete Fourier transform [41]:

(1) $$y\left[h\right]=\overset{n}{\underset{k=1}{\sum }}z\left[k\right]e^{-2\pi i\left(k-1\right)\left(h-1\right)/n}$$

for h=1,…,n where n is the length of the signal z. 

**Figure 6:**
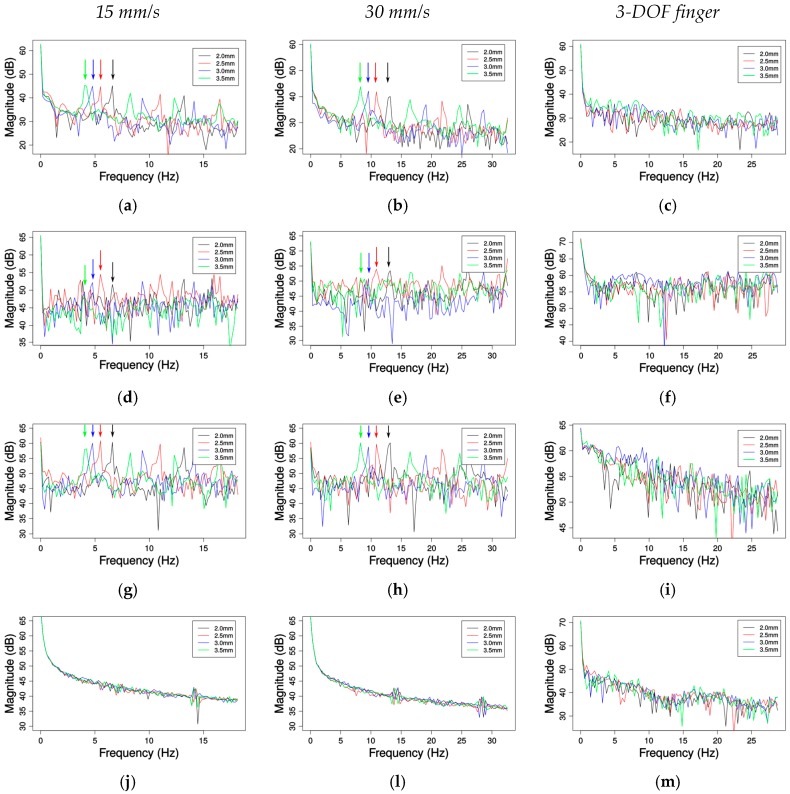
Frequency analysis: (**a**–**c**) deep pressure sensor data; (**d**–**f**) acceleration (*x*-axis); (**g**–**i**) angular velocity (*y*-axis); and (**j**–**m**) magnetic field (*z*-axis).

Figure 6 shows the frequency content of the signals collected by each sensor while exploring the 4 grating patterns in the linear motion and in the robotic finger setup. The first two columns of graphs show the magnitude spectrum in dB of data gathered under 15 and 30 mm/s, respectively. The last column shows the magnitude spectrum in dB for the exploration performed by the robotic finger. The graphs concentrate in frequencies up to 30 Hz, close to the expected frequencies for the grating (Table 3). In all graphs from Figure 6, black lines represent the frequencies of the signals collected over the grating pattern with 2.0 mm groove width; red, blue and green represent the frequencies related to the patterns with a groove width of 2.5 mm, 3.0 mm and 3.5 mm, respectively. The first row of Figure 6 shows the frequencies corresponding to the changes in pressure. The second row shows the frequencies for the changes in acceleration. Third and fourth rows show the frequencies for the data collected by the gyroscope and magnetometer respectively. Regarding the linear motion setup, as expected, the pressure frequencies show peaks (as pointed by the vertical arrows in Figure 6a,b) around 5 Hz for 15 mm/s; the 30 mm/s graph shows that the frequencies are shifted to 10 Hz. The frequencies present in the data gathered by the accelerometer do not present high amplitude differences, but a similar frequency profile can be seen, for example, in the red line with fundamental frequencies and harmonics following the same periods of the pressure changes in Figure 6a,b.

The angular velocity frequencies have the same behavior as the pressure sensed by the deep pressure sensor; the pronounced peaks around 5 Hz in Figure 6g are shifted to around 10 Hz in Figure 6h. The frequencies seen in Figure 6j–l shows that there are no significant changes in the magnetic field orientation and amplitude, except when the metallic linear motion carriage passes under the module and disturbs the magnetic field. As expected the 3.5 mm groove width grating yields the lowest frequencies; the frequencies detected for the 3.0 mm, 2.5 mm and 2.0 mm groove width are ordered from low to high as shown in peaks identified by the arrows in the Figure 6a,b,g,h. The last column of Figure 6 shows that the frequency content of the data collected on the finger setup does not contain peaks around the frequencies expected for each pattern, but localized features on frequency/time domain can be detected by the use of wavelets to characterize surfaces as demonstrated by the experiment in the next section.

Finally, Table 4 compares the expected frequencies for different gratings and velocities from Table 3 (in the row “Expected”) with the detected frequencies by the module (“Mean detected”). The Root Mean Squared (RMS) error is computed as the difference between the expected and detected frequencies while using the linear motion setup, over six passes for each grating pattern and using linear velocity.

The results are close to the expected ones, with a maximum difference of ±0.1 Hz. These errors could be due to small imperfections in the 3D printed gratings patterns, small deviations on the speed controller, and/or to the small variations of the sampling rates.

#### 3.2.2. Experiment 2: Surface Profile Classification

**Figure 7:**
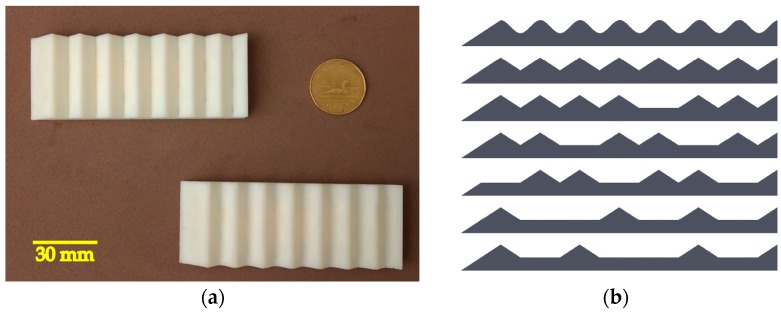
3D printed shapes: (**a**) Shape 2 on the top and Shape 1 on the bottom; and (**b**) list of shapes from Shape 1 on the top to Shape 7 on the bottom.

This experiment expands the previous one by using localized features to distinguish shapes from the data collected throughout an exploratory movement using the same robotic finger from the second setup of the previous experiment. The shapes used in this experiment are coarser than the gratings from the previous experiment. These shapes increase the degree of complexity of the task due to the fact the module may lose contact with their surface. The ABS 3D-printed shapes are fixed in front of the robotic finger (as seen in Figure 4) that establishes contact and slides the tactile tip over them. Shapes 1 and 2 can be seen in Figure 7a, while the seven profiles used for testing are shown in Figure 7b.

A standard recorded movement is executed over each of the shapes and the data representing acceleration, angular velocity, magnetic field intensity and direction, is saved in a database. The database contains 100 runs for each shape, 700 samples in total. From these, 175 samples (25 for each class) are randomly chosen for training, in order to accommodate for tip abrasion, temperature variance, and other possible outliers. Over the time, the tip could become worn and the temperature due to friction could affect the collected data. This process of sampling avoids as well overtraining and increases the generalization capability of the classifier trained to distinguish between patterns.

**Figure 8:**
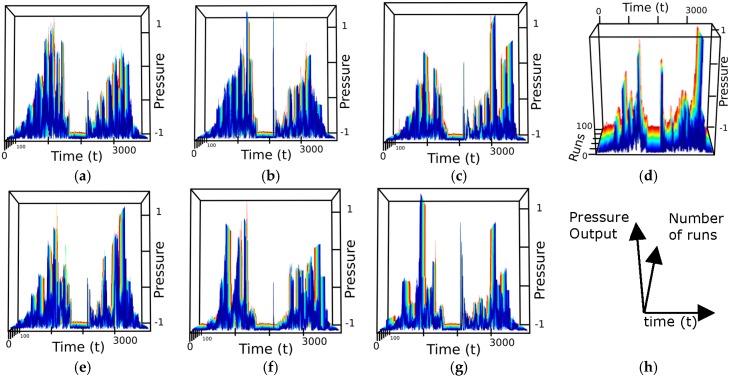
(**a**–**g**) Normalized pressure samples collected over the Shapes 1 to 7; and (**h**) meaning of axes for the preceding plots.

The graphs in Figure 8 show for example the normalized pressure data for each shape. The vertical axes represent the pressure level (Figure 8h), the horizontal axis the discrete time, varying for 0 to 3800 and the number of sample varying from 0 to 100 is shown over the depth dimension. Figure 8d is presented under a slightly different viewpoint to emphasize the number of runs collected.

The data collected pass by three stages to discriminate the surfaces: (*a*) wavelet decomposition; (*b*) principal component analysis; and (*c*) classification by a multilayer perceptron (MLP) neural network.

Initially, the signals of each axis collected by the MARG system and barometer were subjected to a Discrete Wavelet Transform (DWT) decomposition [42] in order to reduce the noise and detect discriminative features of interest. The DWT decomposition consists on the simultaneous filtering of the signals by a low-pass filter, to generate the approximation coefficients, cA1, and by a high-pass to produce the detail coefficients, cD1. The same operation is iteratively applied to the previous approximation signal producing new levels of approximation cAi and detail cDi (i is the level of the decomposition). In this paper, the low-pass and high-pass filters applied to the sensors signals are based on the fifth order of Symlets wavelet (sym5) and the decomposition level is set to 5. The frequencies for the low pass and high pass filters for each level are determined by $\left[\frac{\pi }{2^{i}}\sim \frac{\pi }{2^{i-1}}\right]$ for the high pass filter and $\left[0\sim \frac{\pi }{2^{i}}\right]$ for the low pass filters, where i is the decomposition level. 

The data resulting from the wavelet decomposition are then subject to principal component analysis (PCA). PCA [43] is a multivariate method of analysis widely applied on multidimensional data sets for dimensionality reduction. This approach reduces the number of variables in a dataset retaining as much as possible of its variances. PCA achieves the retention of variance by projecting the original correlated variables into a new set of uncorrelated variables. This reduction is carried out by taking a dataset consisting of p variables X1...Xp and finding the combinations of these to produce uncorrelated orthogonal principal components (PCs) PC1...PCp. There are many ways to find PCs; according to the eigenvalue decomposition, PCs are eigenvectors basis for transformation from data space to feature space. The eigenvalues related to these eigenvectors are a measure of each PC representativeness. Organizing the eigenvalues and eigenvectors enables the selection of the most important principal components. The projection of the original data onto a subset of PCs reduces the dataset dimensionality maintaining the variance proportional to the eigenvalues of the number of PCs used. In this paper, original data of each sensor are reduced to the number of components corresponding to 90% of the total variance.

After PCA is performed on the wavelet decomposition data, we apply a multilayer perceptron neural network to classify the signals into their corresponding profiles. The feature vectors resulting from the PCA are the input to a two-layer feed forward neural network with ten nodes in the hidden layer, and seven neurons in the output layer, the latter corresponding to the number of shapes. The activation functions of all neurons are hyperbolic tangents. The network is trained using the scaled conjugate gradient backpropagation algorithm [44].

The results for the classification of data representing the 5th-wavelet approximation level of each sensor axis are shown in Table 5. 

Each DOF of the MARG sensor is classified individually. The accelerometer group obtains a classification accuracy between 85.1% and 92.6%. The accuracy when considering the gyroscope group is between 93.3% and 98.9%. When considering the magnetic field variations along the trajectory, the results range is 86.9% and 91.4%. The pressure profile measured by the barometer yields an accuracy of 98.9%. The results from the acceleration suggest that the *z*-axis is the least aligned of the three. Because the angular velocity sensor is sensitive to shock/vibration even when the direction of the event is not aligned with its axis, it obtains high accuracy rates in all axes. The low variations of the magnetic field lead to classification rates not as high as those obtained by the inertial sensors. The classification using the data measured by the barometer achieved one of the highest accuracy rates due to the variation of pressure over time and discontinuity of the contact between tactile tip and shapes while the movement was performed. As expected, the best axis of each sensor combined show the highest accuracy. We consider that in a larger dataset with more shapes and complex movements the system would require more components to achieve satisfactory rates of classification. Removing more than 10% of the principal components could narrow data representativeness and generalization. Having redundant measurements may make the system more robust in case of a failure of one of such axes, or in environments and tasks that are not favorable to one or more of such axes (e.g., in the presence of unexpected ferromagnetic objects).

**Figure 9:**
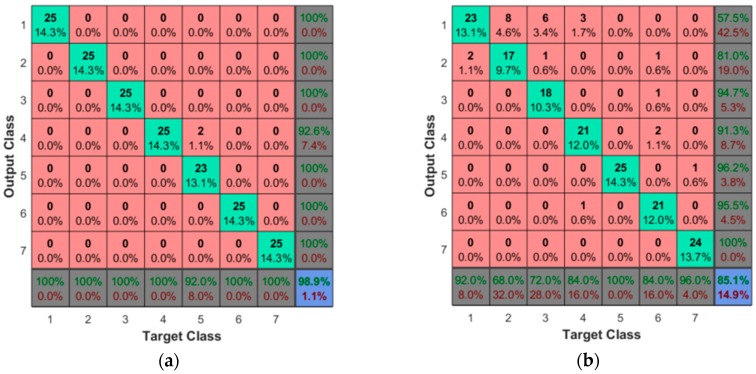
Confusion tables for: (**a**) barometer, showing the misclassification of Shape 5 as Shape 4; and (**b**) accelerometer on *x*-axis, showing the misclassification between Shapes 1 and 2.

Most of the classification errors for the inertial sensors happened between Shapes 1 and 2 due to their similarity, as shown in Figure 8b and in the confusion table in Figure 9b. This is not the case when the sensor considered is the barometer, as shown in Figure 9a where the misclassification mainly affects Shape 5.

## 4. Conclusions

This paper presented a novel bio-inspired design for a tactile module considering the mechanoreceptors’ placement and function and the skin hardness. The compliant structure embedding the sensor allows for vibration of the MARG unit and guides forces acting on the tip area to the deep pressure sensor.

The module is tested in two experiments to study the sensors response to ridged surfaces and coarser shapes. The first experiment showed the traditional sensors in the module can detect fundamental frequencies, varying from 3.66 Hz to 11.54 Hz with an overall maximum error of ±0.1 Hz, over grating patterns sliding under the module at a velocity of 15 mm/s and 30 mm/s, respectively. This experiment also shows that such frequencies are dependent on constant orientation, velocity and pressures that are not detected in a 3-DOF robotic finger setup where such variables are not kept constant. The second experiment showed how localized features in the data collected over seven coarser shapes by sensing module while it was attached to the end of the robotic finger can be classified. The measurements were subject to wavelet decomposition and the 5th level approximation had its dimensionality reduced by 10% before the classification by a neural network. Classification accuracies from 85.1% to 98.9% for the various sensor types demonstrate the usefulness of traditional MEMS as tactile sensors embedded into flexible substrates.

The placement of sensors in the design presented in this paper focuses on dynamic simulation tasks. In future works, the module’s structure should be modified to accommodate the study of static stimulation tasks as well. The future work will also concentrate on the evaluation of the proposed approach over a larger dataset, including fabrics and plastics for texture classification and on how different wavelet decomposition levels can be employed to discriminate finer texture.

## Figures and Tables

**Table 1:** Human skin mechanoreceptors.

Receptor	Properties and Functions
Merkel disks	Location: Shallow Receptive field: small (2–3 mm)—Frequency Range: 0–100 Hz (Peak at 5 Hz) Functions: form/shape detection; texture detection, fine details discrimination, constant pressure, presence, location and static deformation at points and edges; curvature detection; tactile flow perception.
Ruffini corpuscles	Location: Deep Receptive field: large (larger than 10 mm)—Frequency range: 0–? Hz (Peak at 0.5 Hz) Functions: directional (lateral) skin stretch; direction of object motion; position of hand and fingers; slip detection; stable grasp; tangential force estimation; tension (continuous touch or pressure).
Meissner’s corpuscles	Location: Shallow Receptive field: small (3–5 mm)—Frequency range: 1–300 Hz (Peak at 50 Hz) Functions: motion detection; velocity; slippage; low frequency vibration; local skin deformation; tactile flow perception; grip control; flutter; dynamic deformation; two-point discrimination; encode normal (horizontal) strain forces.
Pacinian corpuscles	Location: Deep Receptive field: large (larger than 20 mm)—Frequency range: 5–1000 Hz (Peak at 200 Hz) Functions: deep pressure; pressure change; un-localized high frequency vibration; body contact when grasping an object (tool use).

**Table 2:** LSM9DS0 and MPL115A2 frequency range and sensor resolution [37,38], *Selected M. range* and *Selected F. range*, are the Measurement range and Frequency range the sensors are configured to operate at.

	Magnetic Sensor	Gravity Sensor	Angular Velocity	Pressure Sensor
*Measur. range*	±2–±12 Gs	±2–±16 g	±245–±2000 dps	50–115 kPa
*Selected M. range*	±2 Gs	±2 g	±245 dps	*Not applicable*
*Frequency range*	3.125 Hz–100 Hz	3.125 Hz–1600 Hz	3.125 Hz–1600 Hz	333.3 Hz (max)
*Selected F. range*	100 Hz	800 Hz	800 Hz	333.3 Hz
*Resolution*	0.08 mG	0.061 mg	8.75 mdps	0.15 kPa

**Table 3:** Expected frequencies for different gratings and velocities.

f=v/Δp	Δp=4.1 mm	Δp=3.6 mm	Δp=3.1 mm	Δp=2.6 mm
v=15 mm/s	3.66 Hz	4.17 Hz	4.84 Hz	5.77 Hz
v=30 mm/s	7.32 Hz	8.33 Hz	9.68 Hz	11.54 Hz

**Table 4:** Root Mean Square (RMS) error for six runs of each grating.

f=v/Δp		Δp=4.1 mm	Δp=3.6 mm	Δp=3.1 mm	Δp=2.6 mm
v=15 mm/s	Expected	3.66 Hz	4.17 Hz	4.84 Hz	5.77 Hz
	Mean detected	3.66 Hz	4.10 Hz	4.83 Hz	5.86 Hz
	RMS error	0	0.07	0.01	−0.09
v=30 mm/s	Expected	7.32 Hz	8.33 Hz	9.68 Hz	11.54 Hz
	Mean detected	7.32 Hz	8.35 Hz	9.67 Hz	11.44 Hz
	RMS error	0	−0.02	0.01	0.10

**Table 5:** Classification results.

Sensor	Accuracy (%)
Accelerometer X	92
Accelerometer Y	92.6
Accelerometer Z	85.1
Gyroscope X	98.3
Gyroscope Y	93.3
Gyroscope Z	98.9
Magnetometer X	88
Magnetometer Y	86.9
Magnetometer Z	91.4
Barometer	98.9
Acc. Y—Gyro. Z—Magn. Z—Barometer	100
